# Advanced Generation Seed Orchard of *Abies alba* Mill. in Romania Combining Genetic Gain and Diversity

**DOI:** 10.3390/plants15111603

**Published:** 2026-05-23

**Authors:** Georgeta Mihai, Alin-Madalin Alexandru, Maria Teodosiu, Emanuel Stoica, Paula Garbacea, Lavinia Ifrim

**Affiliations:** 1 Department of Forest Genetics and Tree Breeding, “Marin Drăcea” National Institute for Research and Development in Forestry, 077190 Bucharest, Romania; gmihai_2008@yahoo.com (G.M.); paula.garbacea@icas.ro (P.G.); lavinia.ifrim@icas.ro (L.I.); 2 “Marin Drăcea” National Institute for Research and Development in Forestry, Câmpulung Moldovenesc Station, 73 bis, Calea Bucovinei, 725100 Câmpulung Moldovenesc, Romania; teodosiumaria@yahoo.com

**Keywords:** silver fir, genetic diversity, genetic parameters, breeding strategy, seed orchard, second-generation seed orchard

## Abstract

The genetic parameters at 6, 9 and 12 years were studied in two progeny trials (one half-sib and one full-sib) of silver fir (*Abies alba* Mill.) in Romania, in order to establish an appropriate breeding strategy for advancing second-generation seed orchards. The half-sib trial (HS) consists of 60 open-pollinated families of plus trees from four first-generation seed orchards, while the full-sib trial (FS) consists of 51 half-diallel crosses of 11 plus trees from one seed orchard. Tree height and diameter were found to be under moderate to strong genetic control at both the family and individual levels. Total height showed a higher percentage of additive genetic variance than diameter in both types of progenies. Additive genetic variances increased with age for the diameter (from 12% to 36%), while for the total height, it decreased (from 76% to 35%). In the HS trial, family heritability was higher than individual heritability for both traits. The highest values of heritability were obtained for total height, both at the individual (0.76–0.35) and family levels (0.88–0.63). In FS progenies, the estimates of the narrow-sense individual heritability were lower than those at the family level and remained almost constant over time. The additive age-age genetic correlations and genetic correlations among growth traits were more stable and stronger in FS progeny than in HS progenies. Expected genetic gains were calculated at individual and family levels for different breeding strategies. The highest genetic gain will be obtained through selection of the best parents. Genetic gain slightly varied over age and for progeny tests. The level of genetic diversity, calculated for selected parents based on the breeding values, was high, while the inbreeding coefficient reduced. Combining the backward selection strategy with SSR analyses allows optimization for seed orchard design in order to mitigate inbreeding depression risks and enhance genetic diversity in the next breeding generation.

## 1. Introduction

Silver fir is a monoecious and wind-pollinated, generally outcrossing species. The outcrossing rate in silver fir is over 80% of all seeds produced, similar to many other conifer species. However, in small populations and some years with poor flowering, self-fertilization can occur up to 95% of the produced seeds [1]. Studies carried out by Kormutak and Lindgren showed that the self-fertilization rates in silver fir populations ranged from 8 to 54%, which demonstrates that inbreeding is an important component of its mating system [2]. Teodosiu et al. [3], using seven nuclear microsatellite markers across 36 populations from the Romanian Carpathians, reported inbreeding coefficient values ranging from −0.072 to 0.081 (*p* < 0.001).

Silver fir is characterized by low genetic variability among populations but high genetic diversity within populations, even in marginal populations [4,5], which could be a benefit for adapting to climate warming [6]. In the Romanian Carpathians, representing the edge of species’ distribution, genetic differentiation among populations is relatively low, while high genetic diversity, both allelic richness and expected heterozygosity (He = 0.779–0.834 and AR = 11.61–14.93), has been reported within populations [3]. The level of genetic diversity of all populations from the Eastern Carpathians is higher compared to those previously reported for silver fir in Europe [7,8,9]. Thus, marginal populations represent valuable genetic resources for breeding programs.

Tree breeding programs represent long-term investments aimed at improving the economic and social value of forests. These programs may involve both conventional and modern breeding methods, including genetic selection, field-test designs, mating designs, pedigree information, and genomic tools, with the objective of developing advanced breeding generations with improved characteristics related to wood yield, quality, and resistance to biotic and abiotic stresses. Depending on the objectives of the breeding program, as well as available resources and the genetic base, various breeding strategies have been developed and applied in forest species to maximize genetic gain per unit of cost and time [10,11,12,13].

A breeding strategy can be defined as a combination of breeding methods to achieve specific objectives [14]. In advanced-generation breeding, improving genetic gain while preserving gene diversity represents a primary objective. To establish the most appropriate species breeding strategy, several factors must be considered, including the species’ biology, the genetic variation and parameters of the desirable traits, the choice of selection method, the avoidance of inbreeding, and the maintenance of genetic diversity. All of these factors influence the genetic gain achieved in advanced-generation breeding.

Key parameters for the success of a breeding strategy for any species are heritability and age–age genetic correlations [15]. Heritability provides information on the inheritance of traits important for selection, particularly the relative contributions of additive and dominance genetic variance, while age–age genetic correlations are essential for determining the optimal selection age that maximizes genetic gain per year within a breeding program [16,17,18,19]. Early selection at the juvenile stage can reduce generation time and increase annual genetic gain [20]. However, genetic parameters for many forest tree species remain poorly known due to their long rotation cycles. Consequently, mature performance is often predicted based on assessments conducted in juvenile field trials. For silver fir, despite its silvicultural importance, even fewer data are available regarding the genetic parameters of key traits [21,22,23,24,25,26].

In Romania, the silver fir breeding was initiated approximately 50 years ago, with the main objectives of improving productivity, wood quality, and adaptability to environmental conditions. To enhance the genetic quality of the forest reproductive material, 11 clonal seed orchards, covering a total area of 95 ha, were established in the 1980s. The silver fir first-generation seed orchards comprise 550 vegetative copies of untested plus trees selected in natural populations and were established by the National Institute for Research and Development in Forestry. Currently, these seed orchards are over 45 years old; therefore, advancement to second-generation seed orchards has become necessary.

To address these challenges, an advanced-generation breeding program for silver fir has been developed since 2007. To evaluate the breeding value of the plus trees from first-generation seed orchards and to define an appropriate breeding strategy, eight progeny tests based on both half-diallel and open-pollination families were established between 2014 and 2018. These trials included 83 first-generation parent-trees, 83 open-pollinated families derived from plus trees in five seed orchards, and 109 full-sib families from plus trees in two seed orchards that were tested.

Aspects related to the design of the advanced-generation seed orchards aimed at maximizing genetic and economic gains have been widely discussed in the literature. Thus, selection is more efficient in those traits with high amounts of additive genetic variation [27]. Selection can be conducted at the individual tree level, the family level, or through a combination of both. Assuming random mating, results show that parental selection always offers more genetic gain than offspring selection [28,29]. Advanced-generation orchards may consist only of the offspring selected from progeny tests (forward selections), initial parent genotypes (backward selections), or mixtures of both approaches [30].

Other important aspects related to advanced-generation seed orchards include the number of component clones, the degree of relatedness among them, and orchard design to minimize inbreeding. The recommended numbers of unrelated clones should range between 25 and 40 clones [27,31,32,33], while randomized orchard designs are generally considered most appropriate. Randomized designs, by producing different, non-repeating neighbor patterns, will help reduce the risk of inbreeding depression resulting from selfing. Candidate clones for the new orchard will be those with the highest breeding value estimates. Including the best clones in higher frequencies, the genetic gain can increase compared to equal representation of clones [28,34,35]. Therefore, well-designed seed orchards will maximize genetic gain and genetic diversity, contributing to long-term breeding objectives and improving the genetic quality and adaptability of forests [29].

In this study, we propose a breeding strategy for silver fir aimed to advancing to second-generation seed orchards, based on half-sib and full-sib progenies produced from first-generation seed orchards. Combining breeding approach with genetic tools will represent a significant step forward in the application of modern breeding strategies for this species and opening new perspectives for genetic improvement, adaptability, and sustainable forest management.

This study aimed to: (1) estimate the genetic parameters for growth traits based on performance in half-sib and full-sib progenies; (2) estimate the age-age genetic correlation; (3) calculate and compare the expected genetic gains in progeny tests under different selection strategies; (4) calculate the parental breeding value; (5) select the best parental genotypes for the second-generation seed orchard; and (6) determine the genetic diversity, relatedness among candidates, and the level of inbreeding in the planned seed orchard using SSR markers.

## 2. Results

### 2.1. Genetic Parameters in Half-Sib and Full-Sib Progenies

The estimates of the genetic parameters in the HS trial are listed in Table 1. The additive genetic variance (σ^2^_a_) to the total phenotypic variance varied depending on the trait and age. Total height obtained greater values of additive genetic variance compared with root collar diameter at the age of 6, and the percentages of the additive variance from phenotypic variance are similar for the two traits at the age of 9 (25% and 23%) and 12 (35% and 36%). Additive genetic variances increased with age for the diameter from 12 to 36%, while for the total height it decreased from 76 to 35%.

Proportion of additive genetic variance in phenotypic variance is given in brackets, σ^2^_a_ additive genetic variance, h^2^_ns_i_ narrow-sense individual heritability, h^2^_ns_HS_ half-sib family heritability.

The family heritability values were higher than the individual heritability values for both traits. The highest values of heritability were obtained for total height both at the individual and family level (between 0.76 and 0.35 for h^2^_ns_i_ and 0.88 and 0.63 for h^2^_ns_HS_). Both family (h^2^_ns-HS_) and individual heritabilities (h^2^_ns-i_) had the same trend over time with the additive genetic variances, decreasing for the total height (h^2^_ns_HS_ between 0.88 and 0.63 while h^2^_ns_i_ between 0.76 and 0.35) and increasing for the diameter (h^2^_ns_HS_ between 0.50 and 0.55 while h^2^_ns_i_ between 0.12 and 0.36) (Table 1).

In the FS trial, the additive genetic variance was smaller compared with the HS trial for both traits (Table 2). The additive genetic variance was greater for the total height than for the root collar diameter, ranging from 50 to 24% for total height and 31 to 39% for diameter. For the studied period, the additive genetic variance of total height decreased with age, while for diameter was relatively stable. Narrow-sense FS family heritability increased with age and recorded close values for both traits. Narrow-sense HS family heritability had the same trend as the narrow-sense FS family heritability, having slightly higher values and very close to each other for both traits. The estimates of the narrow-sense individual heritability were smaller than those at the family level and were almost constant over time. For both traits, the values of the individual heritability were very close and appropriate to the values of individual heritability in HS progenies. The individual heritability within FS families (h^2^_n**s-w**_) recorded the lowest values, ranging between 0.11 and 0.22. The ratio of nonadditive variance to additive variance for total height decreased from 4 at age 6 to 2.5 at age 12.

### 2.2. Genetic Correlations Among Traits

**Figure 1:**
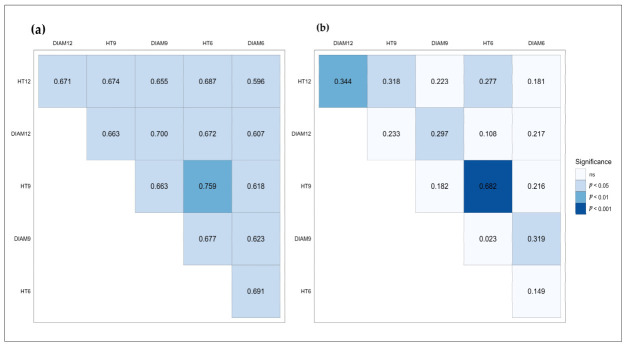
Genetic correlations among traits in Comanesti FS trial (**a**) and in Sacele HS trial (**b**).

In HS progenies, the genetic correlations between growth traits during the measurement period were positive but less significant (Figure 1b). Age-age genetic correlations for total height were high and significant, while for diameter, significant correlations were obtained between DIAM12 and DIAM 9, and between DIAM 9 and DIAM 6. All genetic correlations decreased over time in HS progenies.

The genetic correlations between traits and age-age genetic correlations for total height and root collar diameter in FS progenies are shown in Figure 1a. The additive age-age genetic correlations and genetic correlations among growth traits were stable and stronger than those obtained for HS progenies.

### 2.3. Expected Genetic Gain Under Different Selection Strategies

The genetic gains were calculated both at the individual and family levels for different breeding strategies: individual or mass selection, individual selection for clonal deployment, backward selection on half-sib family mean, forward selection on full-sib family mean or the best progeny within crosses. Expected genetic gains were calculated as the percentage increase in the selected genotypes or families over the population mean.

For the HS progenies, the expected genetic gain from individual mass selection was lower than the genetic gain from narrow-sense family selection (Table 3). The selection of the best individuals within families will increase genetic gain compared with the family’s selection. The highest genetic gain for growth traits will be obtained through the selection of the best parents from first-generation seed orchards. In this case, depending on selection intensity, a possible genetic gain of 19–16% for total height and 22–18% for diameter will be obtained in the next breeding cycle of silver fir. Expected genetic gains varied among the assessment ages. Regardless of the selection method, the percent gains decreased from age 6 to age 12 for height and increased for diameter, respectively. At the same selection intensity (20%), the height gains decreased from age 6 to age 12, with 30% through family and parents’ selections and with 26% through selection of individuals within families. Genetic gains for diameter at age 12 were over 140% higher than at age 6, for all selection methods.

For the FS progenies, the individual mass selection brought the lowest genetic gain (Table 4). The FS family selection will bring a greater genetic gain than the individual selection within families for both traits. The highest genetic gain will be obtained through the selection of the best parents. There is a slight variation over age for diameter, while the total height genetic gains remained almost constant.

Expected genetic gains did not vary greatly among progeny tests, slightly higher values being obtained in FS progenies. Height differences between the HS progeny and FS progeny were greatest at age 9, while for diameter, at age 6, both for the parents’ selection.

Genetic gains for the total height and diameter realized in the first-generation silver fir seed orchards at different assessment ages are presented in Table 5. Realized gains have been calculated as the average performance of open-pollinated seedlings in the progeny test in comparison with the performance of seedlings from natural populations (unimproved material). Overall, the genetic gain of improved material performed significantly better than the unimproved material, with realized gain between 17 and 6% for total height and 5 and 4% for diameter, depending on the age. Comparing the results from Table 3 and Table 4 with those from Table 5, the second-generation seed orchard will bring a significantly higher growth gain than the first-generation seed orchards. Differences in average gain between them were over 200% higher for HT12 and 400% higher for DIAM12.

### 2.4. The Parental Breeding Value Estimates and Selection of the Best Parental Genotypes

Selection of the candidate genotypes for the second-generation seed orchard was made through backward selection of the best parents from progeny tests. The breeding population analyzed in this study comprise 71 silver fir parent-trees from first-generation seed orchards originating from 11 natural populations. To avoid inbreeding in the second-generation seed orchard, a sub-lining scheme was used. According to the origin of the plus trees, the breeding population was divided into 11 groups. The genetic value of each parent was estimated based on its breeding value (BV) calculated in Sacele HS and Comanesti FS progeny tests.

The establishment location of the planned second-generation seed orchard will be in the B1 provenance region, and the candidate parents will be selected from the same and the adjacent provenance regions, with appropriate climate conditions. Considering that the number of 30 component clones will ensure an adequate level of genetic diversity, the top parents from seven breeding groups were selected. The number of selected parents per breeding group was not equal because the size of the groups was not equal, and some parents were superior to others. Between 2 and 10 individuals from each breeding group were selected.

**Figure 2:**
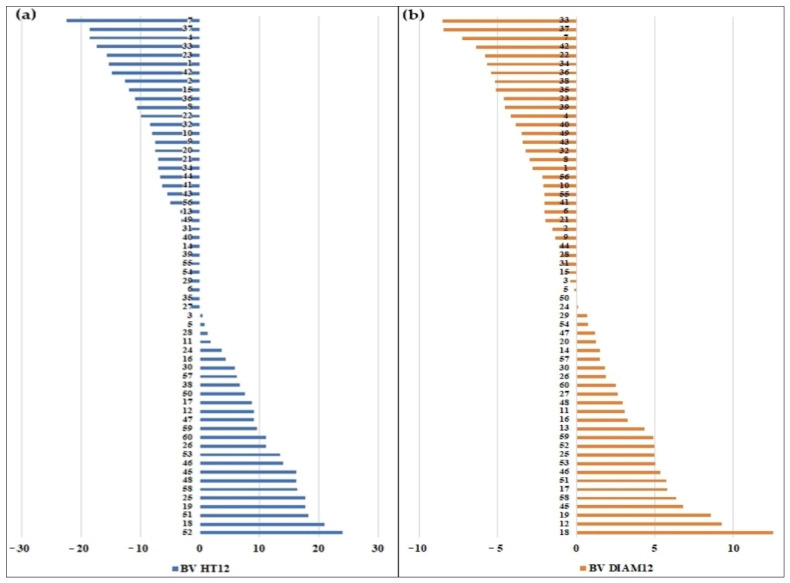
The breeding values of the parents were tested in the Sacele HS trial. Total height (**a**) and diameter (**b**).

The ranking of the parents based on their breeding values for HT12 and DIAM12 calculated in the Sacele HS trial is presented in Figure 2. Selecting the best 26 parents out of 60 (43%) based on their BV will result in a genetic gain of 12% for H12 and 14% for DIAM12.

**Figure 3:**
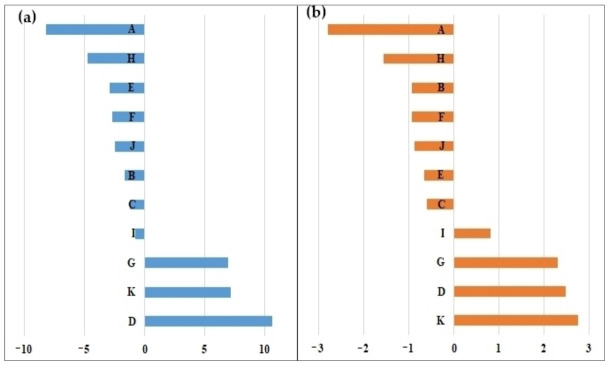
The general combining ability effects of the parents tested in the Comanesti FS trial. Total height (**a**) and diameter (**b**).

The general combining ability effects (GCAs) of each parent included in the Comanesti FS trial are presented in Figure 3. Both positive and negative significant effects (*p* < 0.05) were found for HT12 and DIAM12. The highest positive GCA effects for both traits were obtained by the parents D, K, G and I, while the parents A and H showed the highest negative GCA effects. Therefore, the parents D, K, G, and I will be selected for the next-generation seed orchard, which will bring a genetic gain of 15% in height and 16% in diameter.

### 2.5. Genetic Diversity of the Planned Second-Generation Seed Orchard

The 13 microsatellites (7 nSSR and 6 EST-SSR) showed a polymorphism rate of 92.31%, the Aat06 locus was monomorphic for all individuals and was excluded from subsequent analyses. For the 12 microsatellites, a total of 118 alleles were identified. The number of alleles per locus ranged from 2 (Aat15) to 20 (Sf78). Expected heterozygosity (He) varied between 0.064 (Aat15) and 0.923 (NFF7), while observed heterozygosity (Ho) varied between 0.067 (Aat15) and 0.967 (NFF7) (Table 6). The Polymorphism Information Content (PIC) was closely correlated with heterozygosity levels, reaching its maximum at the NFF7 locus. Across all 12 SSR loci, the average PIC was 0.626, indicating a moderate discriminatory power. The fixation index (FIS) ranged from −0.123 at the Aag01 locus to 0.018 at the NFF3 locus, respectively. Overall, the 30 selected parents exhibited a moderate level of genetic diversity, with a mean number of alleles (Na) and an effective number of alleles (Ne) per locus of 9.66 and 5.36, respectively. The average values of Ho and He were 0.681 and 0.653, respectively, and the mean value and Shannon’s information index (I) equaled 1.59. The fixation index was FIS = −0.041, indicating a slight excess of heterozygotes compared to the expected proportions under Hardy–Weinberg. The genotypic data revealed that each of the 30 individuals showed a unique profile, allowing for the genetic fingerprinting of the selected parents.

**Figure 4:**
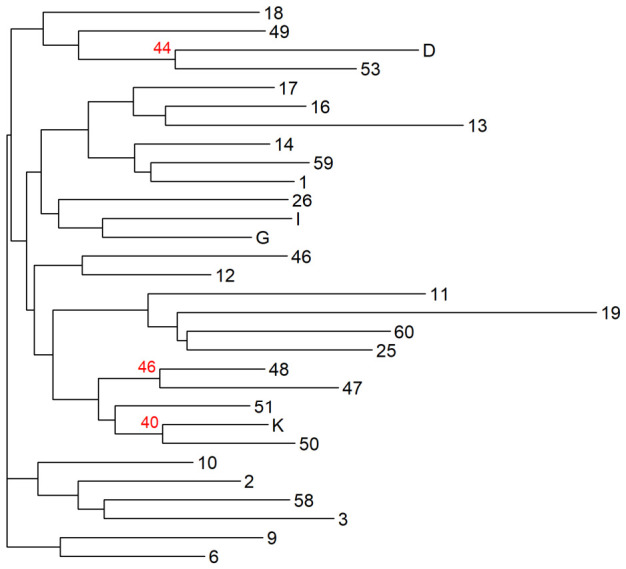
Neighbor-Joining dendrogram based on genetic distances among the 30 selected parents. Bootstrap support greater than 40% with 999 permutations calculated using Nei’s genetic distance it is highlighted in red above the branch.

The Neighbor-Joining (NJ) dendrograms revealed a lack of well-defined hierarchical clustering among the 30 selected parents, indicating a highly homogeneous genetic structure within the studied group (only three sub-clusters have a bootstrap support of over 40%) (Figure 4). Similarly, the PCoA plot showed a scattered distribution of individuals without the formation of distinct, separated clusters. The first two principal coordinates explained a cumulative variation of 19.6%, further suggesting that genetic variation is distributed continuously rather than in discrete groups (Figure S1, Supplementary Materials).

**Figure 5:**
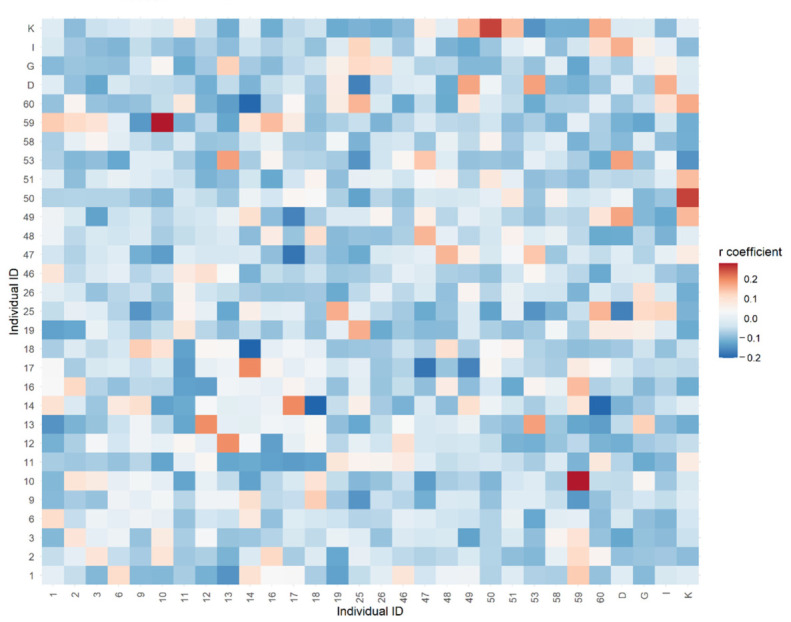
Genetic relatedness between 30 selected silver fir parents based on 12 SSRs markers.

The relatedness coefficient among 30 selected parents obtained with SSR ranged from −0.208 to 0.293, on average of −0.034. Among all pairs of selected parents, two pairs (59–10 and 25-I) had a relatedness coefficient higher than 0.250. Additionally, 16 pairs had a relatedness coefficient over 0.125, respectively. The values over 0.250 suggest a kinship level equivalent to half-siblings. The 16 pairs (3.7% of the total) with a value over 0.125 indicate that some selected individuals originate from the same natural families. The heatmap based on the relationship coefficient matrix is broadly consistent with the Neighbor-Joining dendrogram (Figure 5).

## 3. Discussion

In this study, data from the silver fir first-generation progeny trials were used to establish the strategy for advancing to second-generation seed orchards. Both full-sib and half-sib designs provide good estimates of the genetic parameters and parents’ general combining ability. The open-pollinated design involves lower costs compared with other designs (i.e., full-sib), while half-diallel provides nearly as much information as the full diallel but at a greatly reduced cost. 

Silver fir, like many forest species, is characterized by long rotation cycles, which makes breeding extremely expensive and time-consuming. Thus, to shorten the breeding cycle, early-age selection is a common practice in the breeding program of many forest species. Determining the values of the genetic parameters and their trend with age is very important for establishing the optimal age for early selection and the breeding strategy. 

Growth traits are considered the most economically valuable traits in breeding, as they directly affect wood yield and economic gain. Therefore, in this first stage, selection was based solely on growth traits following that in the next breeding stages to integrate others characters such as adaptive traits. In our study, genetic parameter estimates for growth traits did not vary greatly among progeny tests, slightly higher values being obtained in FS progenies. The variation pattern over time was similar in HS and FS progenies; however, genetic variation was slightly higher in HS progenies than in FS progenies. Also, total height displayed a larger variation with age and higher genetic control compared with diameter, indicating that more genetic gain in terms of tree volume can be expected by the selection for height. 

The results reported in the literature regarding genetic parameters of forest trees and their trends over the years are very different. Hodge and White [28] reported a high heritability for height at early ages in slash pine that decreased after age 10. For Scots pine (*Pinus sylvestris*), Haapanen [36] found heritability almost constant over age. Heritability of height at different ages in loblolly pine (*Pinus taeda*) was found to reach a maximum between ages 14 and 16 years [37]. In the Baltic region, heritabilities for growth and stem quality of Scots pine and Norway spruce varied from 0.05 to 0.25 [38,39]. In Scandinavia, heritability for growth traits of *Betula pendula* was found to vary from 0.07 to 0.56 up to the age of 10 years, largely depending on the planting site [40]. Lai et al. [41], while examining growth and morphologic traits for *Pinus elliottii* families at 27 years old, found higher estimates of the family heritability than individual heritability, and higher value for height than for diameter. Despite the economic importance of silver fir, there are still very few results regarding genetic parameters and genetic gain [26,42].

Predicted genetic gains for the second-generation seed orchard presented in the current study are large and significantly higher compared with those realized in first-generation seed orchards. Among the analyzed selection methods, parental selection is expected to provide the greatest genetic gain at age 12 (16–18% for HT12, 18–19% for DIAM12), suggesting that improvement in the next breeding cycle of silver fir is ensured both from economic and genetic points of view. The expected genetic gain values were very appropriate for the two types of progenies. The decrease in expected genetic gain in the HS progeny trial over time could be explained by imperfect age-age genetic correlations. Similar results were reported for ponderosa pine by Hamilton and Rehfeldt [43] and Joo et al. [44] highlighted that although the relative gain decreased over time, the absolute gain in cubic meters increased over time, being 38% higher at a rotation age of 50 years.

Results regarding the genetic gains obtained from the first-generation of silver fir seed orchards in Romania are comparable with those reported for other coniferous species. Carson et al. [45] reported realized gains of 4.5% in height, 6% in diameter, and 15% in stem volume at ages 15–17 in radiata pine (*Pinus radiata*) seed lots from open-pollinated seed orchards. Realized genetic gains from the first-generation improvement program of Douglas-fir in the North Oregon were about 6% for height, 8% for diameter, and 28% for stem volume [46]. Lundströmer et al. [47] reported a genetic gain of 9–15% in *Picea abies* height and diameter from seed orchards compared with local unimproved stands. Samuel and Johnstone [48] found a genetic gain for Scots pine, at age 10, between 8 and 12% in height and 0 and 3% in stem form compared with unimproved material.

The genetic gain achieved from second-generation seed orchards of *Picea abies* in Sweden is between 10–15%, while for *Pinus sylvestris* it ranges between 10 and 25%. For the third-generation, the estimated gain is about 25% [49]. For loblolly pine (*Pinus taeda*), second-generation seed orchards realized gains in rotation volume ranging from 13% to 21% [32]. The newest advanced-generation orchards are predicted to have volume gains that exceeding 35% [50]. Realized gains for stand per-hectare volume and mean growth rate in Douglas-fir were at least twice as high in the elite population as in the intermediate population [51].

In breeding programs of many species, reducing the breeding cycle is necessary to improve the genetic quality of seed orchards and to ensure that the next generation of breeding is more efficient. Although many studies have demonstrated that the forward selection is one of the most important advancements in breeding strategy [52], in species that reach sexual maturity at older ages, such as silver fir, backward selection may be more appropriate. For our purposes, to minimize the time to commercial production of seed and maximize genetic gain, the new-generation seed orchard will be based on parental selection, as ramets are physiologically mature and flower earlier compared with seed orchards established based on offspring selection. The results showed that selection around age 12 years would be efficient for advancing to the next breeding generation of silver fir. While gain might be higher with later selection, the added time costs a full breeding cycle. Age 12 appears a good trade-off between maximizing genetic gain and the time costs associated with a full breeding cycle. The early selection of the growth traits at the 6 to 12 year old was also confirmed on other species, being reported in the associated literatures [53,54,55,56,57,58]. However, we must specify the limitations of this study, such as few tests and only the growth characters were analyzed. Subsequent selections should consider other traits such as phenology and adaptive traits, and multi-site experiments.

The genetic diversity of forest trees is fundamental for long-term ecosystem resilience and adaptation to environmental stress. While first-generation seed orchards maintain high diversity through broad phenotypic selection from unrelated populations, the design of advanced-generation orchards is more complex due to inbreeding and co-ancestry. In this study, the genetic diversity parameters obtained for the planned seed orchard were slightly higher than those obtained for the first five-generation seed orchards of silver fir in Romania [59], in terms of the effective number of alleles, the Shannon index, and heterozygosity levels (Ne = 4.91, I = 1.45, Ho = 0.600, He = 0.600, respectively). Also, genetic diversity is comparable to that observed in natural populations [3,60]. The observed genetic homogeneity is consistent with the evolutionary history and reproductive biology of silver fir. High rates of gene flow, driven by wind-mediated pollen dispersal, typically maintain low levels of differentiation among individuals in this species. The presence of divergent outliers within an otherwise homogeneous group is of particular interest for the management of the seed orchard. These individuals likely carry rare alleles that are not frequent in the broader population and in the context of a seed orchard, these outliers are highly valuable, as they contribute significantly to the overall allelic richness and ensure that the resulting seed lots maintain a broad genetic base, which is crucial for the adaptive potential of future forest stands.

In advanced-generation seed orchards, it is essential to implement appropriate seed orchard designs to manage pedigree relationships among selected parental clones. The genetic structure and relatedness among the selected clones indicate that, overall, the selected parents for the new seed orchard are unrelated. This is a favorable result, as it will lead to an increase in genetic diversity and will minimizes the risk of inbreeding depression in the next generation.

## 4. Materials and Method

### 4.1. Genetic Material, Experimental Design, and Assessments

**Figure 6:**
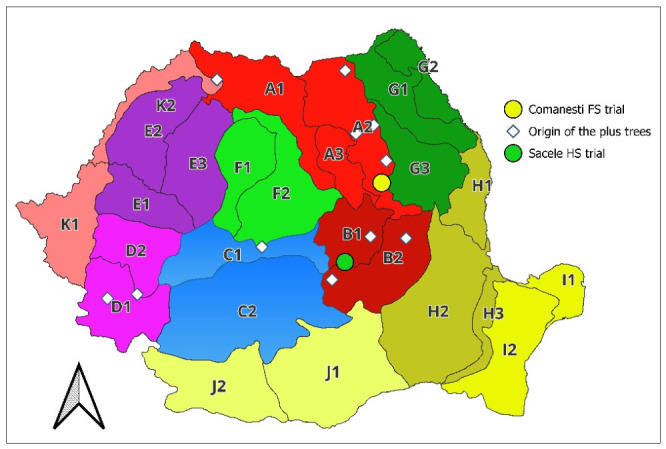
Location of the genetic material and experiments. Provenance regions (A–K) by Pârnuță et al. [61].

Two progeny trials were analyzed in this study: a half-sib (HS, open-pollinated) trial located in the Sacele Forest District and a full-sib (FS, half-diallel) trial located in the Comnești Forest District (Figure 6).

The HS trial was established in 2017 and comprises 144 open-pollinated families from 12 natural populations and 60 open-pollinated families of plus trees from four first-generation seed orchards (Avrig, Tălișoara, Baia Sprie and Gârcina), which originate from 6 natural populations and 6 provenance regions of silver fir in Romania. The FS trial was established in 2014 and comprises 51 half-diallel crosses of 11-plus trees, tested in one first-generation seed orchard (Siminicea), and originated from 7 natural populations and 4 provenance regions.

The field design for the Sacele HS trial was a randomized complete block design with five replications, each with 60 open-pollinated families and 6-seedlings line plots. For the Comanesti FS trial, the design was a randomized complete block design with four replications, each comprising 51 full-sib families (excluding reciprocals and selfs) with 15-seedlings line plots. Both HS and FS trials were planted at 2 m × 2 m spacing with 6-year-old seedlings produced in the same nursery (Sinaia nursery, 695 m above sea level, in the mountain beech zone, at 45°29′ N latitude and 25°59′ E longitude). Survival rate of families ranges from 95 to 100% in HS trial and from 92 to 100% in FS trial.

The data analyzed in this study were total height (HT) and collar root diameter (DIAM) at ages 6, 9, and 12 years (HT6, HT9, HT12, DIAM6, DIAM9, and DIAM12, respectively).

### 4.2. Statistical Analysis

#### 4.2.1. Half-Sib Progeny Test Analysis

Analysis of variance was based on individual seedling measurements using the following linear model [62] for the studied traits:

*Y*_*ijlm*_ = *μ* + *P*_*l*_ + *B*_*i*_ + *F*_*j*_ + (*F* × *B*) _*ji*_ + *e*_*ijlm*_ (1)

where *Y_ijlm_* = performance of the *m*-th seedling from the *l*-th population, *j*-th family, and *i*-th replication; *μ* = overall mean; *P_l_* = effect of the *l*-th population; *Bi* = effect of the *i*-th repetition; *F_j_* = effect of the *j*-th family; *(F × B) _ji_* = interaction between the *j*-th family and the *i*-th repetition; *e_ijlm_* = random error associated with the *m*-th seedling.

Variance components for each trait were estimated with the GLM procedure (SPSS version 20), treating every effect as random except repetition (fixed); expected mean squares were then computed in *R* [63]. We considered family effect as random because the trees tested were random samples from the sampled populations and the repetition effect as fixed to estimate the variance components accurately, because the limitations of the progeny test (5 repetitions and only one environment).

The half-sib family heritability (h^2^_ns_HS_) and individual heritability (h^2^_ns_i_) were calculated as follows [62]:

h^2^_ns_HS_ = σ^2^_F_/σ^2^_Ph1_ = σ^2^_F_/(σ^2^_F_ + σ^2^_FB_/r + σ^2^_w_/rn) (2)

h^2^_ns_i_ = σ^2^_A_/σ^2^_Ph2_ = 4 σ^2^_F_/(σ^2^_F_ + σ^2^_FB_ + σ^2^_e_) (3)

where σ^2^_Ph1_ and σ^2^_Ph2_ = phenotypic variances; σ^2^_A_ = additive genetic variance; σ^2^_F_ = family variance; σ^2^_FB_ = family × repetition interaction variance; σ^2^_e_ = error variance, σ^2^_e_ = σ^2^p + σ^2^_w_/n; σ^2^_p_ = variance among plots; σ^2^_w_ = within-plot variance; n = number of seedlings per plot; and r = number of repetitions.

We have considered the open-pollinated families as true half-sib families (σ^2^_F_ = ¼ σ^2^_A_) because the results of genetic analyses based on 13 nSSR markers in the studied seed orchards indicated a very low level of inbreeding (F_IS_ = 0.003 − 0.030) [59]. Also, to reduce the possible inbreeding and maternal effects [64] in progeny experiments, the collection of seeds for producing offsprings for these trials was done in years with good fructification for silver fir in Romania.

Heritability standard errors were calculated using the Delta method [65].

The estimates of genetic gains were calculated both at the individual (ΔG1), family (ΔG2) and parental (ΔG3) levels for different breeding strategies: forward selection (ΔG1, ΔG2) and backward selection (ΔG3) [66]:

ΔG1 = i h^2^_ns_i_ σ_Ph2_ (4)

ΔG2 = i h^2^_ns_HS_ σ_Ph1_ (5)

ΔG3 = 2 i h^2^_ns_HS_ σ_Ph1_ (6)

Selection intensity i was 1.713 and 1.376 for half–sib family selection (select top 10% and 20% families), 0.869 and 0.200 for individual selection (select top 10% and 20% individuals in trial), and 1.674 and 1.354 (select top 10% and 20% individuals within each half–sib family) [67]. For parental selection, the selection intensity was 20% (i = 1.376) and 30% (i = 1.142) to fulfill the requirement related to the number of component genotypes in orchard design.

Realized gains in the first-generation silver fir seed orchards have been calculated as the average performance of open-pollinated seedlings from seed orchards, in comparison with the performance of seedlings from natural populations, tested in the Sacele HS trial.

Breeding value (BV) is a measure of the genetic quality of an individual as a parent. Breeding value is the additive component of the genotypic value of an individual or the value transmitted from a parent to its progeny. The breeding value of each parent was expressed as deviations from the population mean [68].

BV_HS_ = 2GCA = 2(x_i_ − μ) (7)

where GCA = the general combining ability, x_i_ = the mean of the parent i, μ = the population mean.

The best linear unbiased prediction (BLUP) procedure based on progeny data was applied to calculate the BV of the parent-trees for the second-generation program. The criteria for selection were HT12 and DIAM12.

The genetic correlation coefficient (r_gxy_) was calculated using the formula [69]:

r_gxy_ = cov(g_xy_)/σ_gx_ × σ_gy_ (8)

where cov(g_xy_) are the additive genetic covariance of x and y trait; σ_gx_ and σ_gy_ are the square root of the product of their additive genetic variances.

#### 4.2.2. Full-Sib Progeny Test Analysis

Half-diallel mating design was used (Method 4—Griffing 1956), considering only direct crossings, reciprocal crosses and self-pollination were excluded. All effects were considered random except repetition (fixed) to increase the accuracy of estimating genetic parameters and breeding values [70].

The statistical analysis used the individual seedling measurements follows the mathematical model:

*X*_*ijk*_ = *μ* + *r*_*k*_ + *g*_*i*_ + *g*_*j*_ + *s*_*ij*_ + *e*_*ijk*_ (9)

where *X_ijk_* = the individual seedling observation; *μ* = the overall mean; *r_k_* = the effect of the *kth* repetition; *g_i_* = the general combining ability effect (GCA) of the *i*-th parent; *gj* = the general combining ability effect (GCA) of the *j-*th parent; *s_ij_* = the specific combining ability effect (SCA) of the *i*-th parent and *j*-th parent, so that *s_ij_* = *s_ji,_* and *eijk* = the random error associated with *ijk*-th seedling.

Analysis of variance, correlation analysis, and estimation of genetic parameters for the studied traits were conducted in the *sommer* R package [71].

According to different selection methods, the heritability was calculated as follows [16,72]:

Broad sense full-sib family mean heritability:

h^2^_bs_FS_ = (2σ^2^_g_ + σ^2^_s_)/σ^2^_Ph1_ = (2σ^2^_g_ + σ^2^_s_)/(2σ^2^_g_ + σ^2^_s_ + σ^2^_p_ /r + σ^2^_w_/rn) (10)

Narrow-sense full-sib family mean heritability:

h^2^_ns_FS_= 2σ^2^_g_/σ^2^_Ph1_ = 2σ^2^_g_/(2σ^2^_g_ + σ^2^_s_ + σ^2^_p_ /r + σ^2^_w_/rn) (11)

Narrow-sense individual heritability:

h^2^_ns_i_= 4σ^2^_g_/σ^2^_Ph2_ = 4σ^2^_g_/(2σ^2^_g_ + σ^2^_s_ + σ^2^_p_ + σ^2^_w_) (12)

Narrow-sense individual heritability within family:

h^2^_ns_w_= 2σ^2^_g_/σ^2^_Ph3_ = 2σ^2^_g_/(σ^2^_Ph2_ − σ^2^_Ph1_) (13)

Narrow-sense half-sib family mean heritability:

h^2^_ns_HS_= σ^2^_g_/σ^2^_Ph3_ = σ^2^_g_/[(pσ^2^_g_ + σ^2^_s_ + σ^2^_p_ /r + σ^2^_w_ /r(p − 1)]/p − 1 (14)

where σ^2^_Ph1,2,3_ = the phenotypic variances, σ^2^_g_ = the genetic variance due to general combining ability, σ^2^_s_ = the genetic variance due to specific combining ability, σ^2^_p_ = the variance among plot, σ^2^_w_ = the within-plot error variance, r = the number of blocks, p = the number of parents, n = the number of seedlings within each plot for each cross.

Standard errors (SE) of variance components were estimated using the delta method proposed by Lynch and Walsh [65] in the *msm* R package [73].

The estimates of genetic gains were calculated both at the families and individual level for different breeding strategies [66,74]:

The mass selection genetic gain when they are vegetative propagated:

∆G1 = h^2^_bs_FS_ iσ_Ph1_ (15)

The genetic gain from full-sib family selection:

∆G2 = h^2^_ns_FS_ iσ_Ph1_ (16)

The genetic gain from half-sib family selection:

∆G3 = h^2^_ns_HS_ iσ_Ph3_ (17)

The genetic gain from selection among randomly seedlings in the trial site:

∆G4 = h^2^_ns_i_ iσ_Ph2_ (18)

The genetic gain from selection among randomly placed seedlings within a family:

∆G5 = h^2^_ns_W_ iσ_Ph3_ (19)

The genetic gain from the parents of the first-generation orchards:

∆G6 = 2 h^2^_ns_FS_ iσ_Ph1_ (20)

Selection intensity was i = 1.705 and i = 1.372 for full–sib family selection (select top 10% and 20% families), i = 0.235 and i = 0.007 for individual selection (select top 10% and 20% individuals in trial), i = 1.556 and i = 1.237 for individual selection within family (select top 10% and 20% individuals within each family), and i = 1.324 and i = 1.126 for the parental selection (the best 20% and 30% parents) [67].

General combining ability effects (GCAs) of each parent were estimated following Griffing [43]. Differences between GCA effects were compared using the Student’s “*t*-test”.

Genetic correlations were calculated from the GCA covariance component of two characters in the numerator and the square root of the product of their GCA variance components in the denominator [16]:

r_Gxy_ = σ_gxgy_/√(σ^2^_gx_ × σ^2^_gy_) (21)

where X, Y are two traits, σ^2^gx or σ^2^gy is the GCA variance of traits X or Y, and σ_gxgy_ is the GCA covariance.

### 4.3. Genetic Diversity Analysis

#### 4.3.1. Plant Materials, DNA Extraction and Genotyping

The needles were collected from the 30 selected parental clones based on breeding value, from the first-generation seed orchards (Avrig, Tălişoara, Gârcina, and Siminicea). The samples were freeze-dried and genomic DNA was subsequently extracted using the ATMAB method [75]. DNA concentration and quality were determined using a Biophotometer Plus spectrophotometer (Eppendorf, Hamburg, Germany). In order to ensure consistency with other studies conducted on silver fir in Romania [3,59] (13 nuclear microsatellites (nSSR and EST-SSR) were used, namely NFH15, NFH3, NFF3, NFF7 [76], Sf1, Sf78, Sfb4 [77] and, Aag01, Aat01 Aat04, Aat06, Aat11, Aat15 [78]). The polymerase chain reaction (PCR) was performed in a final reaction volume of 7.5 μL, containing 1× Qiagen Multiplex PCR MasterMix (2×), 2 μM of each primer, and ultrapure water. Amplification was carried out using a MiniAmp Plus thermal cycler (Thermo Fisher Scientific, Waltham, MA, USA) under the conditions described in Teodosiu et al. [59]. PCR products were separated on a GenomeLab GeXP Genetic Analysis System (Sciex/Beckman-Coulter, California, USA) and fragment scoring and data analysis were conducted using the GenomeLab Analysis Software ver. 10.2.3 (Beckman Coulter).

#### 4.3.2. Data Analysis

To assess genetic diversity at both the locus and the planned second-generation seed orchard levels, GenALEx 6.5.3 [79] were employed to calculate the following parameters: number of alleles per locus (A), effective number of alleles (Ae), Shannon’s information index (I), observed heterozygosity (Ho), expected heterozygosity (He) and inbreeding coefficient (Fis). CERVUS 3.7 [80] was used to estimate PIC (polymorphic information content). Genetic relationships among selected parental genotypes were analyzed using package v.4.5.2 (R Core Team). A Neighbor-Joining (NJ) dendrograms was constructed based on Nei’s genetic distance [81] with 999 bootstrap replicates using the poppr (v.2.9.8) [82] and ape (v.5.8.1) [83] in R 4.5.2 package. Additionally, a Principal Coordinates Analysis (PCoA) was performed to visualize the genetic distribution of genetic variation and further explore the relationships between individuals. To quantify genetic kinship using SSR data, we calculated pairwise relatedness coefficients based on the moment-based estimator of Wang [84], and LynchRd [85] and one probability estimators, respectively TrioML [86]. These estimators were selected due to their proven efficiency in analyzing highly polymorphic markers such as microsatellites. Lynch and Ritland (LRM) and Wang estimators are particularly robust for multi-allelic data Wang estimator, especially for small samples size, as is our case. Furthermore, the inclusion of the TrioML maximum likelihood estimator provides a more accurate assessment by accounting for potential genotyping errors and null alleles, ensuring a comprehensive evaluation of the genetic relatedness within the seed orchard. This analysis was performed in COANCESTRY [87]. The strong positive correlation (*r =* 0.72) observed between LRM and the other two estimators, indicating a consistent genetic signal across different statistical models. Finally, the relationship matrix based on LRM coefficient was subsequently visualized as a heatmap using the heatmap.2 function (*gplots* package) in R [88].

## 5. Conclusions

This study presents a conceptual framework for optimizing the genetic gain and genetic diversity in the design of an advanced-generation seed orchard for silver fir.

Findings reveal the genetic diversity characteristics of peripheral populations of silver fir and optimizing the selection strategy of the second-generation seed orchard.

Also, the results of this study highlight high heritability, generally strong genetic correlations among traits and with ages, and large genetic gain, supporting a breeding strategy based on backward selection in progeny tests at juvenile age. Selection of the best 30 parents from seven breeding groups at age 12 years will bring genetic gain between 12% and 15% for height and 14% and 16% for diameter in next breeding generation, reducing the length of the breeding cycle and increases the gain per year.

Combining the backward selection strategy with genetic marker analyses can further optimize seed orchard design by mitigating the risk of inbreeding depression and enhancing genetic diversity in the seed crop, thereby ensuring the long-term success of the breeding programs.

The results of the present study complete the knowledge regarding the inheritance and genetic parameters of growth traits of silver fir and are highly relevant for breeding and conservation programs in Europe.

## Figures and Tables

**Table 1:** Genetic parameters for growth traits at different ages for silver fir open-pollinated progenies from seed orchards.

Traits	Mean	σ^2^_a_	h^2^_ns-HS_	h^2^_ns-i_
HT6	52.91 ± 0.74	74.38 (76)	0.88 ± 0.18	0.76 ± 0.03
HT9	59.75 ± 0.30	66.72 (25)	0.54 ± 0.09	0.25 ± 0.02
HT12	103.68 ± 0.71	327.08 (35)	0.63 ± 0.11	0.35 ± 0.02
DIAM6	9.12 ± 0.07	0.43 (12)	0.50 ± 0.06	0.12 ± 0.01
DIAM9	12.35 ± 0.07	2.44 (23)	0.55 ± 0.09	0.23 ± 0.02
DIAM12	31.16 ± 0.26	43.9 (36)	0.55 ± 0.11	0.36 ± 0.02

**Table 2:** Genetic parameters for growth traits at different ages for silver fir full-sib progenies from seed orchards.

Traits	Mean	σ^2^_GCA_	σ^2^_SCA_	σ^2^_a_	h^2^_bs-FS_	h^2^_ns-FS_	h^2^_ns-HS_	h^2^_ns-i_	h^2^_ns-w_
HT6	27.00 ± 0.22	7.62	33.67	30.49 (45)	0.97 ± 0.02	0.31 ± 0.17	0.64 ± 0.15	0.18 ± 0.13	0.13 ± 0.10
HT9	31.58 ± 0.16	12.39	20.97	49.57 (50)	0.98 ± 0.03	0.53 ± 0.16	0.78 ± 0.07	0.31 ± 0.15	0.22 ± 0.13
HT12	72.70 ± 0.47	44.81	110.03	179.23 (24)	0.91 ± 0.05	0.41 ± 0.16	0.72 ± 0.11	0.27 ± 0.17	0.11 ± 0.07
DIAM6	8.80 ± 0.05	0.43	1.10	1.70 (38)	0.94 ± 0.04	0.41 ± 0.17	0.72 ± 0.11	0.22 ± 0.13	0.15 ± 0.10
DIAM9	11.90 ± 0.05	0.66	1.48	2.62 (31)	0.94 ± 0.04	0.44 ± 0.16	0.74 ± 0.10	0.20 ± 0.11	0.13 ± 0.08
DIAM12	21.39 ± 0.11	4.17	7.53	16.67 (39)	0.95 ± 0.04	0.50 ± 0.16	0.77 ± 0.08	0.49 ± 0.28	0.18 ± 0.10

**Table 3:** Genetic gain (%) by selection of the best families, individuals, and parents in the open-pollinated progenies trial.

Traits	Families		Individuals		Individuals Within Family		Parents	
	10%	20%	10%	20%	10%	20%	20%	30%
HT6	17	13	12	2	24	19	27	12
HT9	15	12	14	3	28	22	24	10
HT12	12	9	9	2	17	14	19	16
DIAM6	5	4	2	1	4	3	9	2
DIAM9	7	5	4	1	8	6	11	4
DIAM12	14	11	11	2	21	17	22	18

**Table 4:** Genetic gain (%) by selection of the best families, individuals, and parents in the full-sib progenies trial.

Traits	Families		Individuals		Individuals Within Family		Parents	
	10%	20%	10%	20%	10%	20%	20%	30%
HT6	14	11	2	0.1	8	6	22	18
HT9	19	16	3	0.1	11	9	26	22
HT12	14	12	2	0.1	5	4	21	18
DIAM6	11	9	1	0.0	6	5	17	14
DIAM9	11	9	1	0.1	5	4	15	13
DIAM12	16	13	3	0.1	5	4	22	19

**Table 5:** Genetic gains for total height and diameter from the first-generation seed orchards of silver fir at different assessment ages.

Seed Orchard	Gain for the First-Generation Seed Orchards					
	6 Years		9 Years		12 Years	
	HT6	DIAM6	HT9	DIAM9	HT12	DIAM12
Avrig	9	6	3	3	2	0
Talisoara	16	0	9	3	7	8
Baia Sprie	15	2	5	0	3	0
Garcina	30	12	18	7	13	9
**Average gain**	**17**	**5**	**9**	**3**	**6**	**4**

**Table 6:** Genetic diversity parameters of the 12 SSR loci.

Marker	A	Ae	I	Ho	He	F_IS_	PIC
NFH3	19	8.37	2.52	0.867	0.881	0.016	0.872
NFF3	9	5.38	1.85	0.800	0.814	0.018	0.791
NFF7	18	12.95	2.71	0.967	0.923	−0.048	0.918
NFH15	13	6.56	2.16	0.833	0.848	0.017	0.833
Sf1	4	2.13	0.94	0.533	0.531	−0.005	0.474
Sf78	20	11.61	2.68	0.933	0.914	−0.021	0.908
Sfb4	13	5.62	2.10	0.900	0.822	−0.095	0.807
Aat01	4	2.03	0.90	0.567	0.508	−0.116	0.448
Aag01	7	5.04	1.72	0.900	0.802	−0.123	0.773
Aat15	2	1.07	0.14	0.067	0.064	−0.034	0.062
Aat04	4	1.18	0.36	0.167	0.157	−0.064	0.152
Aat11	3	2.34	0.92	0.633	0.573	−0.106	0.479
Mean	9.66	5.36	1.59	0.681	0.653	−0.047	0.626

## Data Availability

The original contributions presented in this study are included in the article. Further inquiries can be directed to the corresponding authors.

## Supplementary Materials

The following supporting information can be downloaded at: https://www.mdpi.com/article/10.3390/plants15111603/s1, Figure S1: PcoA analysis.
