# Supplementary figures and images for: Advanced Generation Seed Orchard of Abies alba Mill. in Romania Combining Genetic Gain and Diversity
Source: Plants (Basel). 2026 May 23;15(11):1603. doi: 10.3390/plants15111603 (PMC13258873; doi:10.3390/plants15111603)

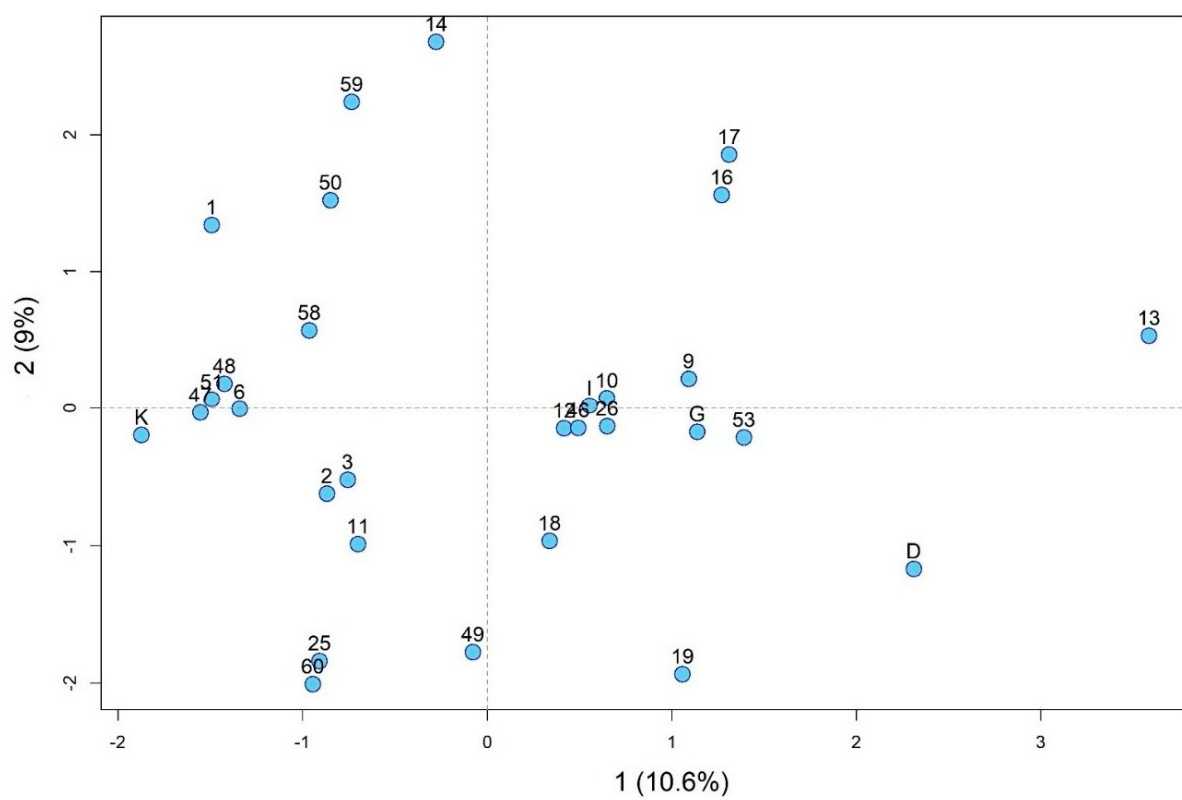

**Figure S1.** PcoA analysis.

Supplement: Supplementary file 1 [file plants-15-01603-s001.zip › plants-4277237-supplementary.pdf]
